# Three-Dimensional Micro-Motion Feature Extraction of the Vibrating Target Based on Multi-Channel Radar in the Terahertz Band

**DOI:** 10.3390/s20010008

**Published:** 2019-12-18

**Authors:** Bin Tang, Qi Yang, Ye Zhang, Bin Deng, Hongqiang Wang

**Affiliations:** College of Electronic Science and Technology, National University of Defense Technology, Changsha 410073, China; tangbin_nudt@163.com (B.T.); fighting_zy10@126.com (Y.Z.); dengbin@nudt.edu.cn (B.D.); oliverwhq@tom.com (H.W.)

**Keywords:** multi-channel radar, terahertz band, three dimensional interference, micro-motion parameters estimation, phase-derived range

## Abstract

Vibration induces a micro-Doppler effect, which contains crucial motion information and features of the micro-motion target. However, the traditional radar can only acquire micro-motion information in the radial direction. The multi-channel radar is capable of acquiring actual micro-motion information in a three-dimensional space, including amplitude, frequency, and three-dimensional direction of vibration. Moreover, the high-accuracy parameter estimation can be achieved in the terahertz band. In this paper, a method for three-dimensional micro-motion feature exaction and parameter estimation is proposed, including a phase-derived-range algorithm, interference, and auto-correlation. Meanwhile, to prove feasibility of the method, the results of simulations and experiment based on a 0.22 THz multi-channel radar system are presented after theoretical analyses.

## 1. Introduction

Vibration, a common type of micro-motion [1], greatly influences radar imaging, thereby resulting in the deteriorated defocusing phenomenon. In terms of equipment development, the vibration may result in harmful resonance. Meanwhile, the motion information and micro-motion features of the vibrating target can also be obtained via radar echo processing. Therefore, estimating the parameters of the vibration is significant for imaging compensation [2,3] and monitoring. However, if the target did not vibrate in the radial direction, the traditional single-channel radar can only acquire the radial component of the echoes. In other words, the micro-motion information acquired is not complete. In addition, the high resolution three-dimensional imaging is sensitive to the slight vibration in the terahertz band. To achieve high resolution imaging of a vibrating target, the three-dimensional micro-motion feature extraction and parameter estimation are extremely necessary. Thus, it is vital to provide complete micro-motion feature via three-dimensional interference of multi-channel radar.

The terahertz (THz) wave refers to electromagnetic wave with frequency between 0.1~10 THz. Its wavelength is approximately 30 um~3 mm, which is much shorter than the wavelength of microwave. Due to its short signal wavelength, terahertz radar is more sensitive to the micro-motion, which means that it can extract more precise micro-motion features. Nevertheless, terahertz radar is also sensitive to the influence of noise. Noise will result in the deterioration of estimation accuracy. Concerned with the current research about micro-motion in three-dimensional space, Y. Luo etc. introduced the multi-input multi-output (MIMO) techniques to the three-dimensional micro-motion feature extraction [4]. Then, D.W. Li etc. utilized netted radars to extract the three-dimensional micro-motion features of a rotating target [5]. D. Zhang etc. studied an extraction method of three-dimensional micro-motion feature from a sliding-type scattering center in ballistic target by utilizing the multi-view characteristics of netted radar [6]. J. Hu and Y. Luo etc. applied the idea of multi-antenna interferometry processing from Interferometric Inverse Synthetic Aperture Radar (InISAR) to the three-dimensional micro-motion feature extraction of ballistic targets [7,8]. J.Q. Wei etc. proposed an algorithm for micro-motion and geometric parameters based on squint calibration via an L formation array [9]. This research all involved precession and rotation micro-motion and exist in a microwave band. In addition, the studies above are all based on simulations in lack of experiments.

In this paper, a three-dimensional micro-motion parameters estimation method for a vibrating target is proposed via multi-channel terahertz radar. The estimated parameters include vibration amplitude, frequency, initial phase, as well as three-dimensional space angles. The vibrating target and radar echo model is established in Section 2. Meanwhile, the method of micro-motion feature extraction and parameters estimation is introduced. The simulation results and performance are presented in Section 3. In Section 4, the multi-channel terahertz radar system and design of experiment are introduced. Then the experimental results and analysis are presented in Section 5. The conclusions are summarized in the last section.

## 2. Theory Analysis

### 2.1. Radar Echo Signal Model of Vibrating Target

Due to the interference processing, a linear frequency modulated (LFM) pulse mode is applied. Assuming that the bandwidth of the transmitted LFM signal was B, carrier frequency was $f_{c}$, pulse width was $T_{p}$, linear frequency modulation slope was γ, the transmitted signal can be expressed as,

(1) $$s(\hat{t},t_{m})=rect(\frac{\hat{t}}{T_{p}})\exp\left(j2\pi (f_{c}t+\frac{1}{2}\gamma {\hat{t}}^{2})\right)$$

where $\hat{t}$ is the fast time, $t_{m}$ is the slow time, and t represents time. The distance between the target and the radar is assumed as R, then the echo signal can be presented as:

(2) $$s_{r}(\hat{t},t_{m})=rect(\frac{\hat{t}-2R/c}{T_{p}})\exp\left(j2\pi (f_{c}(t-2R/c)+\frac{1}{2}\gamma {\left(\hat{t}-2R/c\right)}^{2})\right)$$

Then the distance between the target and the radar is,

(3) $$R=R_{0}+a_{v}\sin(2\pi f_{v}t_{m})$$

where $a_{v}$ and $f_{v}$ are the amplitude, and frequency of vibration, respectively.

After Dechirp processing, the range profile can be expressed as:

(4) $$\begin{array}{l} S_{if}(f_{i},t_{m})=T_{p}\mathrm{sinc}[T_{p}(f_{i}+\frac{2\gamma }{c}(R_{0}-R_{ref}+a_{v}\sin(2\pi f_{v}t_{m})))]•\\ \exp\left(-j\frac{4\pi }{c}f_{c}(R_{0}-R_{ref}+a_{v}\sin(2\pi f_{v}t_{m}))\right) \end{array}$$

Taking advantage of phase information in range profile expression above, which can be written as $\phi_{i}=\frac{4\pi }{c}f_{c}(R_{0}-R_{ref}+a_{v}\sin(2\pi f_{v}t_{m}))$, the range information can be presented as follows,

(5) $$R_{i}=\frac{\lambda }{4\pi }\phi_{i}=R_{0}-R_{ref}+a_{v}\sin(2\pi f_{v}t_{m})$$

where λ is the wavelength of carrier wave. That is the basic principle of phase derived range method.

In addition, the micro-Doppler can be expressed as:

(6) $$f_{d}=\frac{1}{2\pi }\frac{d\phi_{i}(t_{m})}{dt_{m}}=\frac{4\pi }{c}f_{v}f_{c}a_{v}\cos(2\pi f_{v}t_{m}).$$

### 2.2. Phase Derived Range Method

When electromagnetic waves travel a distance of one wavelength, the corresponding phase will change 2π rad. According to this character, we can obtain the expression of phase derived range method as follows,

(7) $$R=\frac{\lambda }{4\pi }\Delta \phi$$

where λ is the wavelength of carrier wave, Δϕ is phase difference between emitted electromagnetic wave and received electromagnetic wave and R refers to the distance between radar and target.

In fact, the estimated phase Δϕ generally varies from 0 rad to 2π rad, which is the result after 2π modulus operating. When the distance between target and radar is above λ/2, the actual phase is not Δϕ unless it is unwrapped. λ/2 is called maximum unambiguous range. After solving ambiguity, the unwrapped phase is the actual phase [10,11], which can be utilized to deduce distance information in Equation (7).

### 2.3. Three-Dimensional Coordinates and Parameters Estimation

Theoretically, the multi-channel radar needs at least 3 receivers to compute the three-dimensional coordinates of target. According to the principle of interference, the three receivers cannot be co-linear. Thus, a reasonable design involves three receivers form a right triangle. The mutually perpendicular receivers can acquire motion information in two vertical directions. Adding the radial direction, the three-dimensional micro-motion information can be deduced.

Actually, three receivers can only acquire a radial micro-motion components and range information. It is difficult to estimate the parameters of three-dimensional micro-motion directly. Furthermore, the distance between receivers and the vibrating target varies with time, and geometric relations are complicated. Therefore, the analytic geometric method is applied to extract parameters [12,13,14].

Establishing an appropriate coordinate system is a vital step. The basic principle of establishing a coordinate system is to avoid introducing irrelevant factors as far as possible. The initial coordinates of the target and three receivers are set as $\left(x_{0},y_{0},z_{0}\right)$, $\left(x_{a},y_{a},z_{a}\right)$, $\left(x_{b},y_{b},z_{b}\right)$, $\left(x_{c},y_{c},z_{c}\right)$. Assuming that the distances from three receivers to the vibrating target are $R_{a}$, $R_{b}$, and $R_{c}$ respectively and the coordinates of vibrating target is (x,y,z), the geometry relationship expressions can be presented as follows:

(8) $$R_{a}^{2}={\left(x-x_{a}\right)}^{2}+{\left(y-y_{a}\right)}^{2}+{\left(z-z_{a}\right)}^{2}$$

(9) $$R_{b}^{2}={\left(x-x_{b}\right)}^{2}+{\left(y-y_{b}\right)}^{2}+{\left(z-z_{b}\right)}^{2}$$

(10) $$R_{c}^{2}={\left(x-x_{c}\right)}^{2}+{\left(y-y_{c}\right)}^{2}+{\left(z-z_{c}\right)}^{2}.$$

The distances $R_{a}$, $R_{b}$, and $R_{c}$ in Equations (8)–(10) can be estimated via phase derived range method. In the above equations, the coordinates of three receivers are set initial values when establishing coordinate system. According to conditions above, the coordinates of the vibrating target (x,y,z) are solved. The coordinates of the vibrating target can be expressed as the sum of vibration components and initial coordinates. Removing the initial coordinates, the estimated vibration components $x_{ve}$, $y_{ve}$, and $z_{ve}$ can be obtained. The vibration component is vital information for estimating three-dimensional micro-motion parameters.

**Figure 1:**
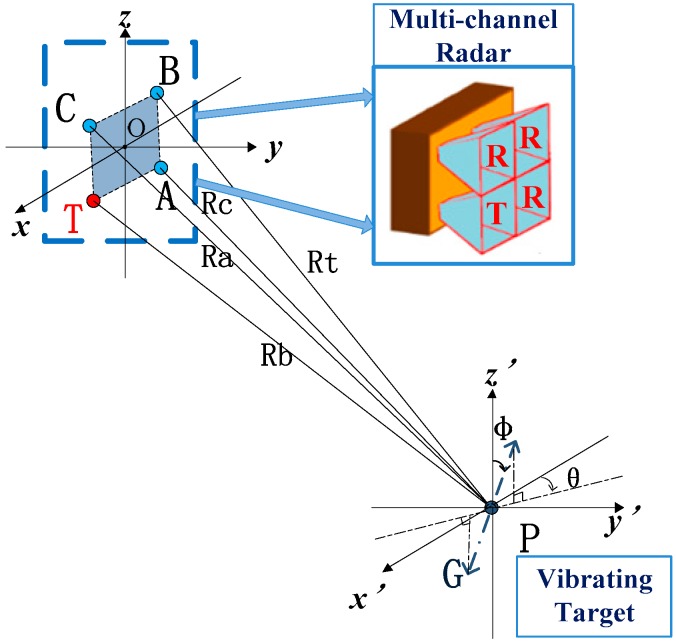
The observation scenario.

Concretely, it is assumed that the multi-channel terahertz radar has 1 transmitting front-end and 3 receivers, which are donated as T, A, B, and C, respectively. The target noted as P is an ideal scatter vibrating back and force along trajectory G. The observation scene is showed in Figure 1.

In Figure 1, θ and ϕ are defined as azimuth angle, and pitch angle, respectively. Theoretically, the expressions of vibration components $x_{v}$, $y_{v}$, and $z_{v}$ should be:

(11) $$x_{v}(t)=R_{v}(t)\sin\phi \cos\theta =A_{v}\sin\phi \cos\theta \sin(2\pi f_{v}t+\varphi_{0})$$

(12) $$y_{v}(t)=R_{v}(t)\sin\phi \sin\theta =A_{v}\sin\phi \sin\theta \sin(2\pi f_{v}t+\varphi_{0})$$

(13) $$z_{v}(t)=R_{v}(t)\cos\phi =A_{v}\cos\phi \sin(2\pi f_{v}t+\varphi_{0}).$$

Therefore, the estimated vibration can be expressed as,

(14) $${\left(R_{ve}(t)\right)}^{2}=(x_{ve}(t){)}^{2}+{\left(y_{ve}(t)\right)}^{2}+{{(z}_{ve}(t))}^{2}=(A_{ve}\sin(2\pi f_{ve}t+\varphi_{0e}){)}^{2}$$

where $R_{ve}(t)$ is the estimated vibration, $x_{ve}(t)$, $y_{ve}(t)$, and $z_{ve}(t)$ are estimated vibration components along the x, y, and z axes.

According to curves and equations above, the parameters of three-dimensional vibration can be estimated, including amplitude, frequency, initial phase, azimuth angle, and pitch angle. As expressed in Equation (14), the data of estimated vibration conforms to sine form. The estimated vibration amplitude and initial phase can be extracted from sine curve directly. As for the estimated frequency of micro-motion $f_{ve}$, the auto-correlation method is applied.

The estimated azimuth angle $\theta_{e}$ and pitch angle $\phi_{e}$ can be computed with estimated components in three directions:

(15) $$\theta_{e}=\arctan(\frac{y_{ve}(i)}{x_{ve}(i)})$$

(16) $$\phi_{e}=\arccos(\frac{z_{ve}(i)}{R_{ve}(i)}).$$

As a result, all the parameters of vibration in three-dimensional directions are estimated. Then, the simulations are carried out to prove the validity of the above theoretical analyses.

## 3. Simulations

### 3.1. The Simulation Design

In simulations, the carrier frequency of multi-channel terahertz radar is set to 0.22 THz and the sample frequency is 2048 Hz. The distance between two adjacent receivers is l=0.02m. As for the vibration, its amplitude, frequency, and initial phase are set to 5 mm, 15 Hz, and 30°, respectively. The azimuth angle θ is π/6 and pitch angle ϕ is π/3. The initial coordinates of three radar receivers and target are set as (l,0,0), (0,0,0), (0,l,0), (0.1,1.2,2). Ideally, the original signal is not affected by the noise. If micro-motion of the target is set as $R_{v}(t)=A_{v}\sin(2\pi f_{v}t+\varphi_{0})$, the coordinates of vibrating target will be $\left(x_{p}(t),y_{p}(t),z_{p}(t)\right)$, where:

(17) $$x_{p}(t)=0.1+R_{v}(t)\sin\phi \cos\theta ,\;y_{p}(t)=1.2+R_{v}(t)\sin\phi \sin\theta ,\;z_{p}(t)=2+R_{v}(t)\cos\phi .$$

**Figure 2:**
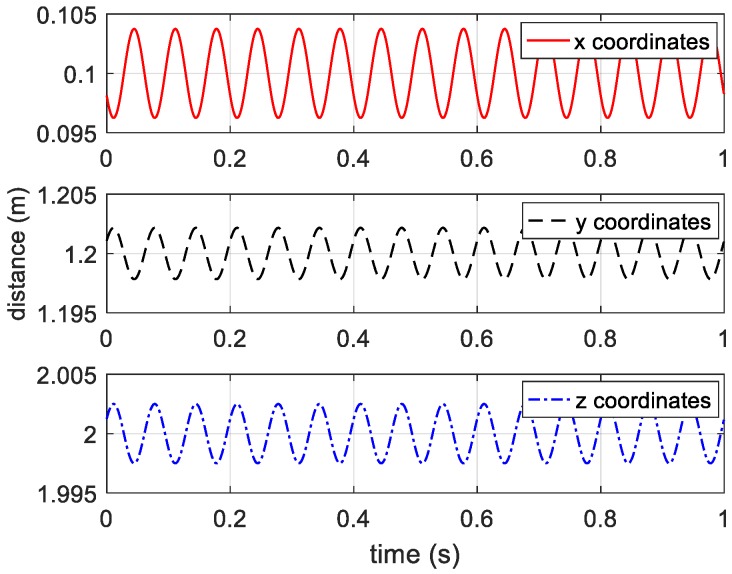
Ideal coordinates of vibrating target.

**Figure 3:**
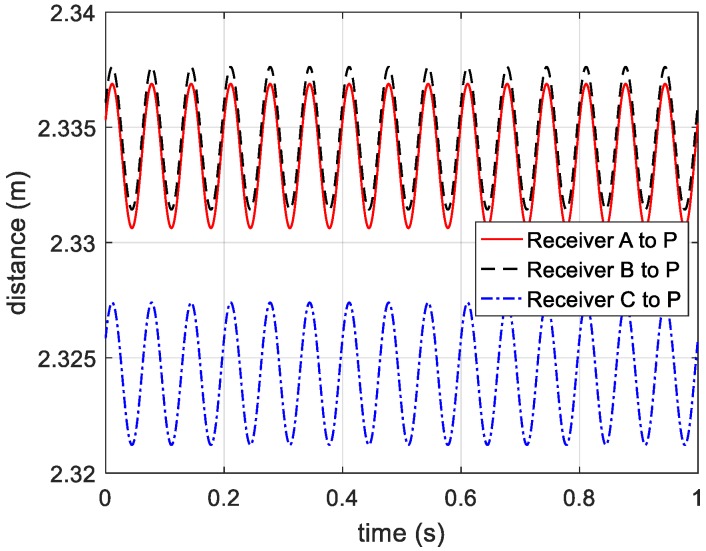
Ideal distance curves between receivers and vibrating target.

In theory, the ideal coordinates and distance curves of vibrating target are shown in Figure 2 and Figure 3, respectively.

### 3.2. The Simulation Results and Performance

**Figure 4:**
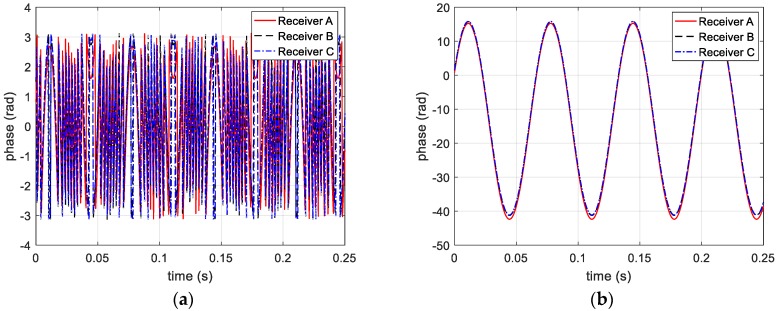
Phase information extracted from three receivers (**a**) before and (**b**) after unwrapped.

To simplify the simulations, a single-frequency signal is applied to the multi-channel terahertz radar. The carrier frequency is 220 GHz, and the pulse repetition frequency (PRF) is 2048. Through extracting phase information from range profile, three phase curves of receivers are shown in Figure 4.

As mentioned above, the distance between target and radar exceeds λ/2 and the actual phase could be acquired after solving ambiguity. Thus, before and after the unwrapping processing, the phase information is shown in Figure 4a,b, respectively.

**Figure 5:**
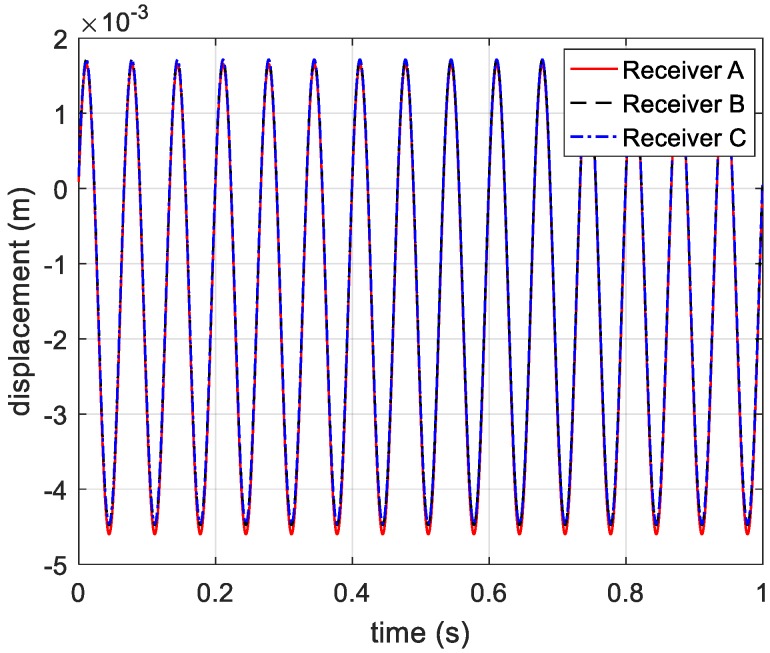
Displacement information acquired from three receivers.

According to the principle of phase-derived range method, the range information expressed in Equation (5) is presented in Figure 5.

**Figure 6:**
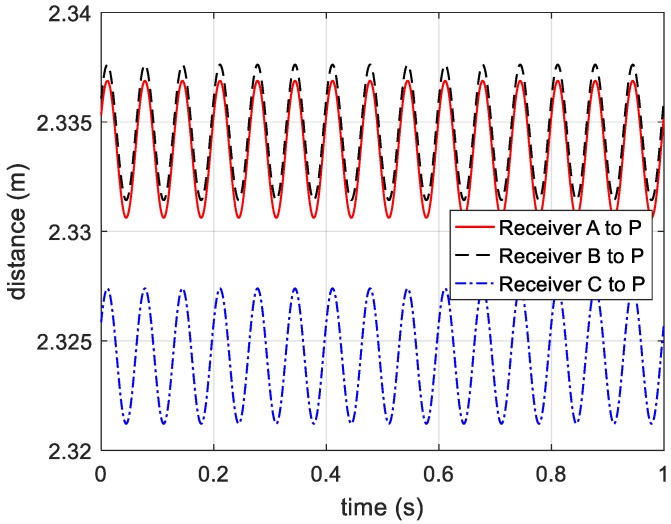
Distance from receivers to the vibrating target.

After compensating the reference distance, the curves of distance from receivers to target are shown in Figure 6.

**Figure 7:**
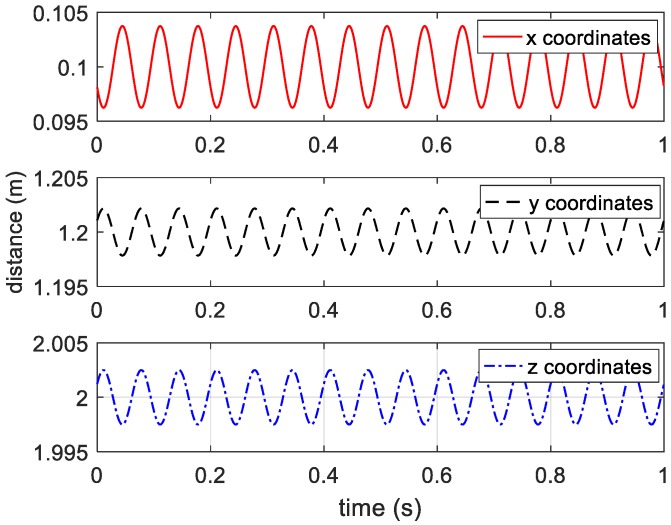
Estimated coordinates of vibrating target.

On the basis of Equations (8)–(10), the estimated three-dimensional coordinates of target are presented in Figure 7.

**Figure 8:**
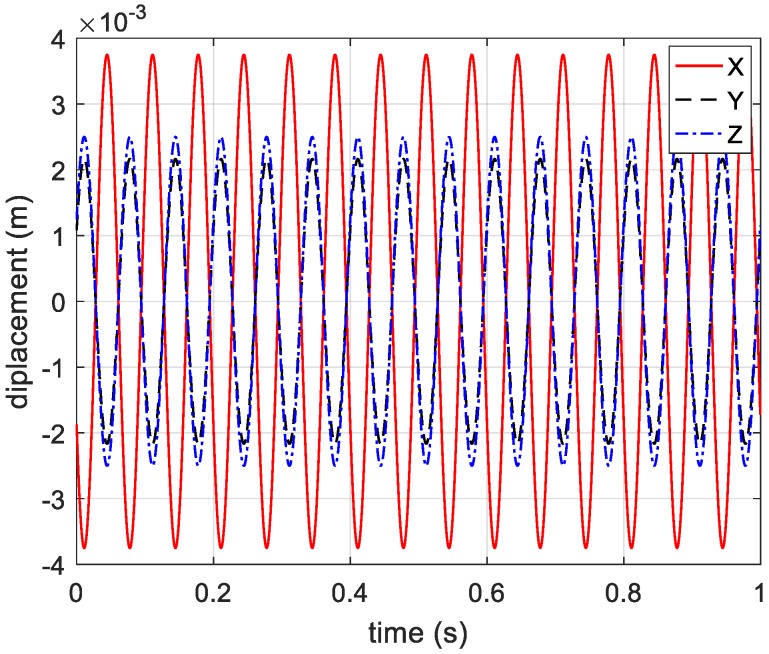
Estimated vibration components in three directions.

Through extracting sine part, the vibration components along the three coordinate axes can be obtained and shown in Figure 8.

**Figure 9:**
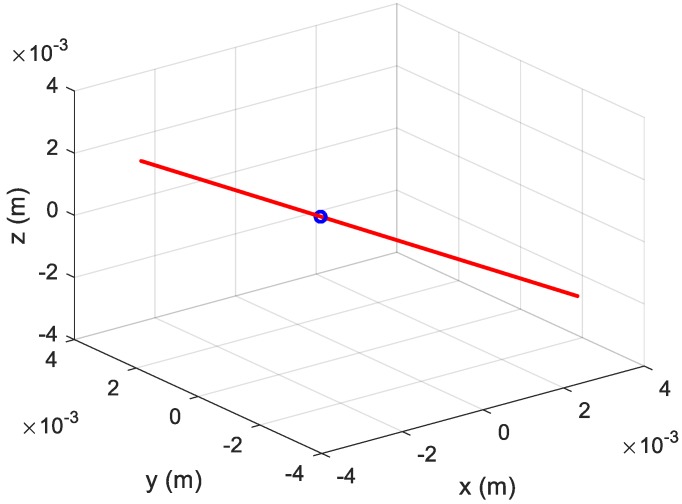
Estimated three-dimensional vibration trajectory.

Combing three estimated curves in Figure 8, the three-dimensional vibration trajectory is shown as a red line and the target is shown as blue point in Figure 9.

Consequently, the estimated parameters are presented in Table 1 as follows.

**Figure 10:**
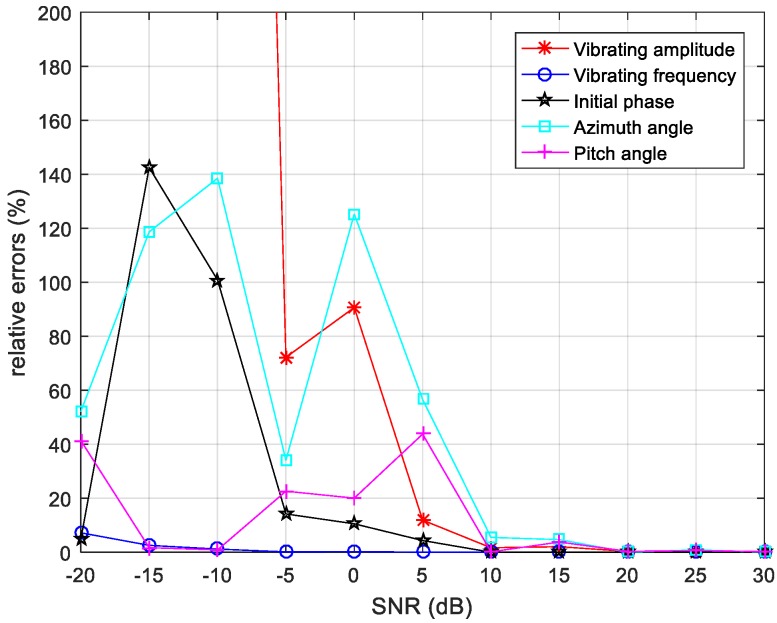
Relative errors of estimated parameters under different signal-to-noise ratio (SNR).

The accuracy and robustness of estimating method are explored. The white Gaussian noise of different signal-to-noise ratio (SNR) is added to the original signal. With the disturbance of noise, the relative errors of estimated parameters are presented in Figure 10.

Although, the estimated results are extremely close to the true values in Table 1, the relative errors fluctuate greatly when adding white Gaussian noise to signals. In Figure 10, the relative errors of all parameters have decreased as SNR increased in size. The relative error of the estimated vibration frequency is lower than 10% with SNR varying from −20 dB to 30 dB. When the SNR is above 10 dB, the estimated parameters are pretty precise. However, the white Gaussian noise causes deterioration of estimation accuracy when the SNR is lower than 5 dB. Due to the randomness of the white Gaussian noise, the fluctuation of relative errors occur, but it is still considered large relative errors.

The reason for the larger error of estimated vibration amplitude re the errors from unwrapped phase information. The noise effects phase of echo signals and the unwrapped phase is inaccurate. According to the Equations (8)–(10) and the initial coordinates of receivers and the target, the coordinates of vibrating target in Equation (17) can also be expressed as:

(18) $$x_{p}(t)=(l^{2}+R_{b}^{2}-R_{a}^{2})/(2l),\;y_{p}(t)=(l^{2}+R_{b}^{2}-R_{c}^{2})/(2l),\;z_{p}(t)=(R_{b}^{2}-x_{p}^{2}-y_{p}^{2}{)}^{1/2}.$$

As is expressed in Equation (18), the x and y coordinates of the vibrating target are relative to the distance between adjacent receivers l. Owing to the short baseline length l, slight deviation of $R_{a}$, $R_{b}$, and $R_{c}$ will cause great errors of vibration amplitude and the three-dimensional space angles. The slight deviation includes amplitude errors and phase shift, which is caused by the unwrapped errors of the phase derived range method. That is the main reason for the relative errors of vibration amplitude and why the three-dimensional space angles are higher than the other parameters.

After a series of analyses, the simulation results prove that the estimation method is sensitive to noise. The main reasons are short baseline length and the limitation of the phase derived range method.

## 4. The Experimental System

### 4.1. The Multi-Channel Terahertz Radar System

The multi-channel terahertz radar system mainly consists of four modules: the signal source, the radio frequency (RF) chains, intermediate frequency (IF) module, and the data collection module. In the signal generator module, the output frequency of local oscillator (LO) is 18.032–18.448 GHz, and the output of RF is 18.092–18.508 GHz. The output power of reference IF signal is approximately 7 dBm, whose frequency is 720 MHz. In the transmitting chain, the IF signal is multiplied into the terahertz band after power amplifying and 12 times frequency multiplications, and the terahertz signal is then transmitted by a conical horn antenna.

**Figure 11:**
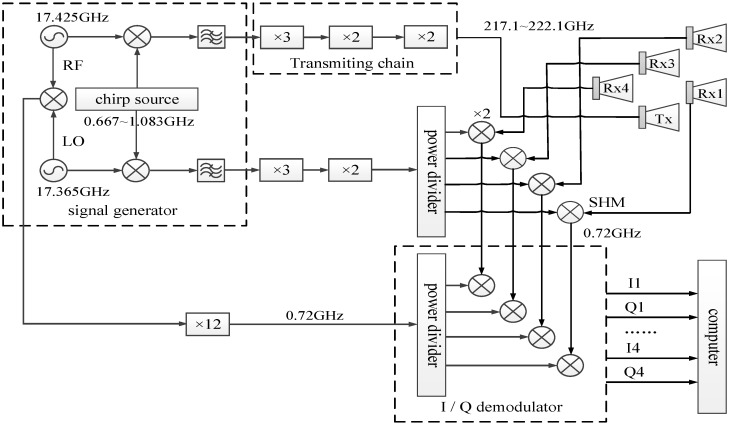
Schematic diagram of the 0.22 THz multi-channel radar system.

**Figure 12:**
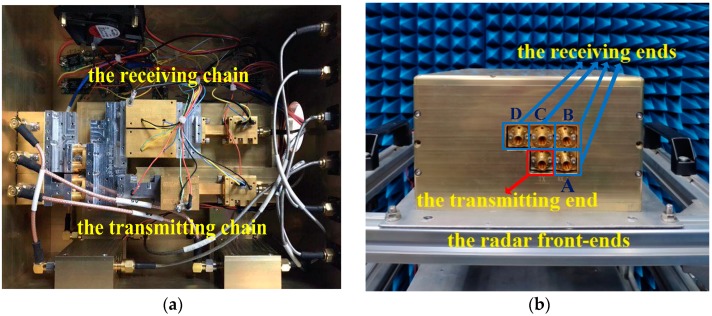
Transmitting and receiving front-ends of the radar system (**a**) inside (**b**) outside.

After sub-harmonic mixer (SHM) processing in the receiving chain, the received terahertz signal is down-converted to baseband and demodulated by an I/Q demodulator for A/D sampling. In the end, Ethernet transmits the data to the computer for further procession. Both transmitting signals and collecting data are controlled by a computer and the control software. The schematic diagram of the 0.22 THz multi-channel radar system is shown in Figure 11. In the system, there are one transmitting antenna and four receiving antennas. The distance between two adjacent front-ends is 2 cm. The L-shaped configuration of receiving antennas is shown in Figure 12, and the four receivers are named front-end A, B, C, and D, respectively.

### 4.2. The Vibration Testing Machine

**Figure 13:**
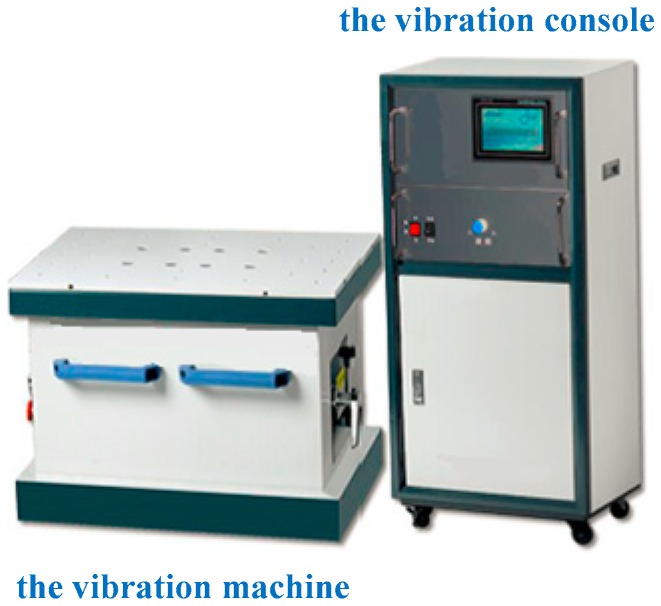
The vibration testing machine.

The vibration testing machine consists of three parts: vibration table, vibration platform, and console. The direction of vibration is vertical, which is produced by the mechanical movement inside the vibration platform. The vibration amplitude and frequency are adjustable, which is controlled by the console. The vibrating frequency ranges from 5 Hz to 55 Hz, and the amplitude is adjustable in the range of 0 mm to 5mm. The vibration testing machine is shown in Figure 13.

### 4.3. The Design of Experiment

**Figure 14:**
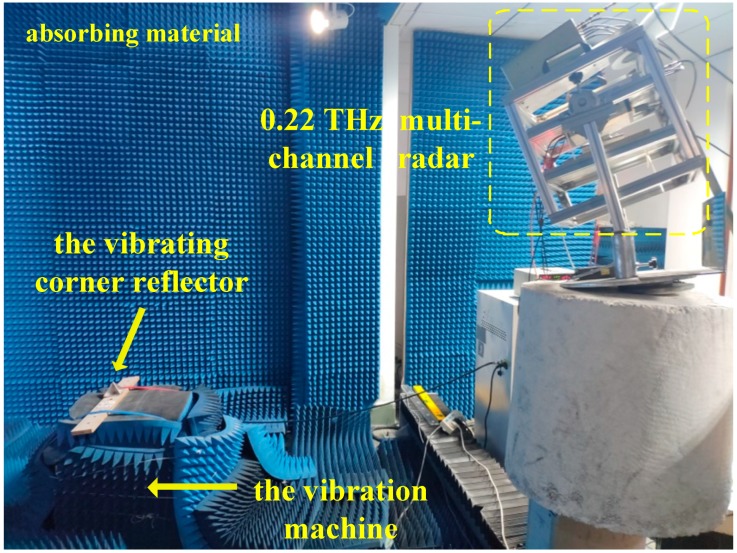
The experimental scenario.

In the experiment, a system, which includes a multi-channel terahertz radar and a vibration testing machine was established. The observed target is a four-sided corner reflector, which is fixed on the vibrating machine to vibrate back and force. To avoid electromagnetic wave reflection caused by the vibration platform, the vibration machine is wrapped with absorbing material. The reference target is the same stationary corner reflector in the initial position. The experimental scenario is shown in Figure 14.

The values of experimental parameters are presented as follows. The carrier frequency of multi-channel terahertz radar is 0.22 THz and the bandwidth is 5 GHz. As for the vibrating corner, its amplitude is set to 5 mm and the vibration frequency is set to 20 Hz. The azimuth angle θ is 80° and pitch angle ϕ is 55°. It is noted that three receivers are enough to estimate the vibration parameters. Additionally, the SNR of front-end C is poor, owing to some issues. Thus, the following experiment only utilize the data acquired from receivers A, B, and D.

## 5. Experimental Results and Estimated Parameters

### 5.1. The Experimental Results

According to the parameters in Section 4.3, an experiment was carried out to verify the feasibility of the method proposed in this paper. The frequency-modulated continuous wave (FMCW) signal is utilized, whose carrier frequency is 0.22 THz and bandwidth is 5 GHz. In the experiment, the frequency-sweep period is 200 μs, and the sampling point of each frequency-sweep period is set to 2048. In addition, the PRF of the signal is set to 2500 Hz.

**Figure 15:**
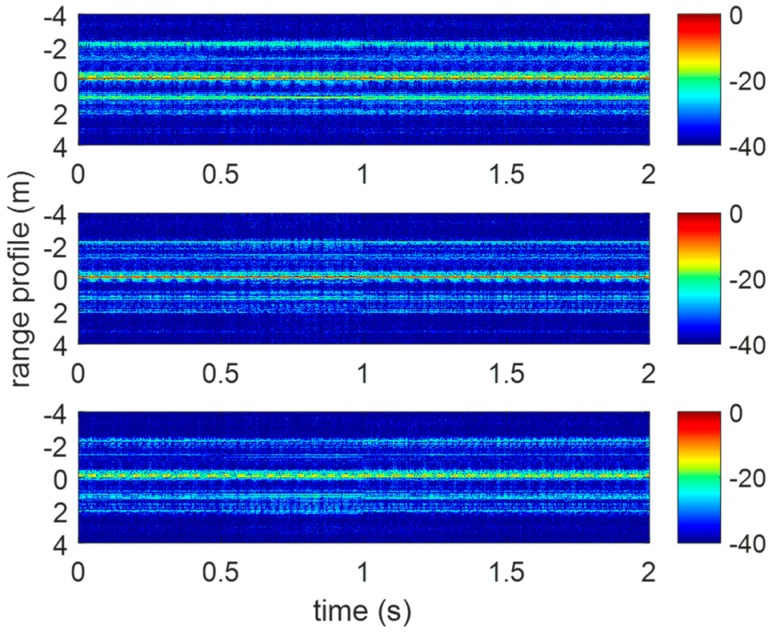
The range profile sequence of three receivers.

**Figure 16:**
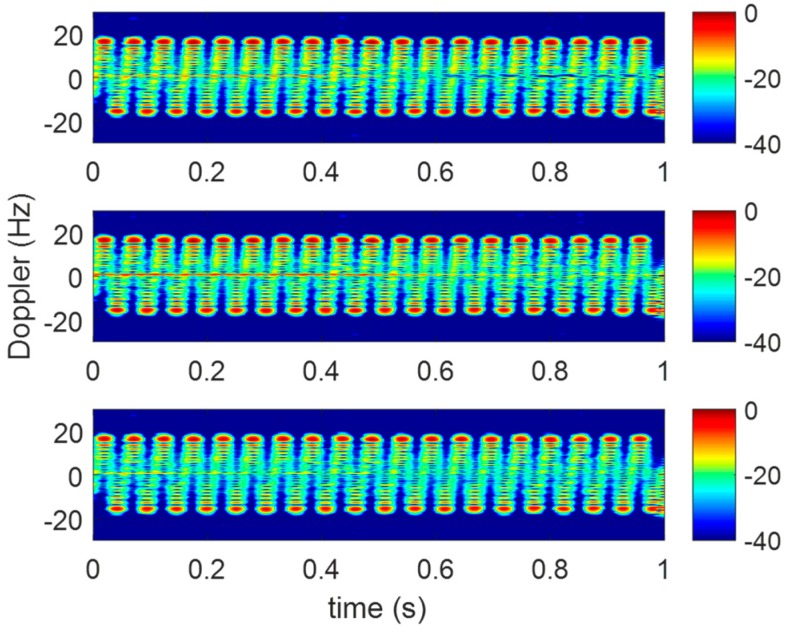
The time-frequency distributions of three receivers.

As is mentioned in Section 4, three receivers are utilized to acquire the returned terahertz signals. The range profile sequences of the vibrating corner reflector are shown in Figure 15. In Figure 16, the micro-Doppler images of three receivers conform to the form of sine curves in Equation (6).

**Figure 17:**
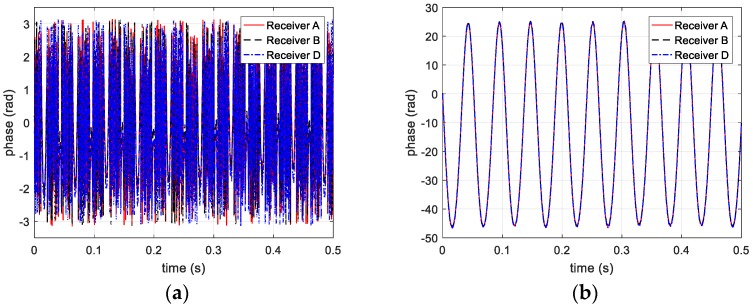
Phase information extracted from A, B, and D receivers (**a**) before and (**b**) after unwrapped.

**Figure 18:**
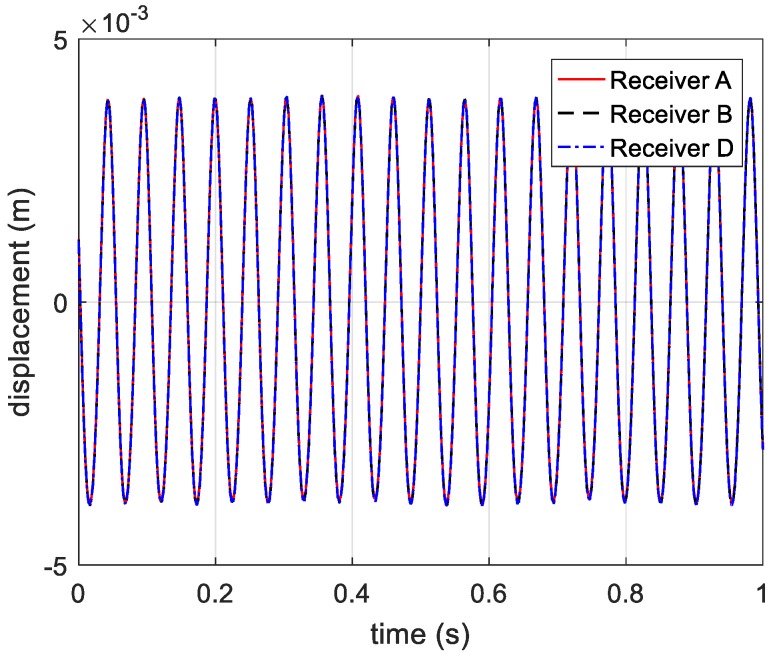
The vibration components in radial directions.

On the basis of analysis in Section 2.2, the phase information extracted from range profile needs ambiguity resolution, which is presented in Figure 17. The vibration components in radial directions are shown in Figure 18. Then, we adopt the phase-derived range method to acquire the distance information from the three receivers to the vibrating corner reflector. The estimated vibration frequency can be obtained via autocorrelation method.

**Figure 19:**
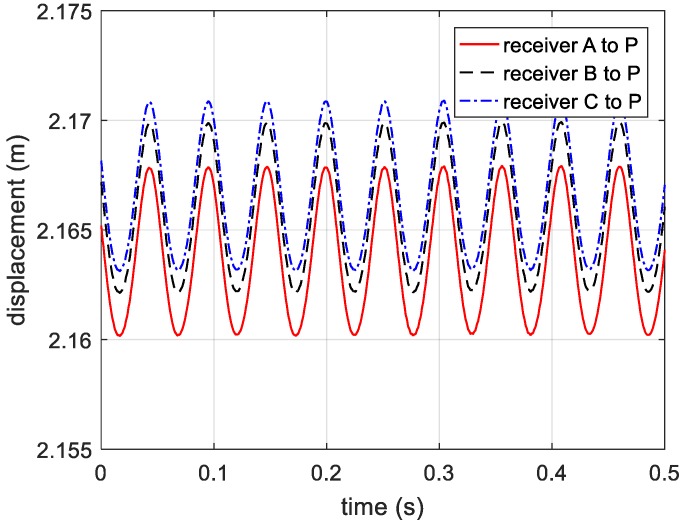
The distance information from three receivers to the target.

**Figure 20:**
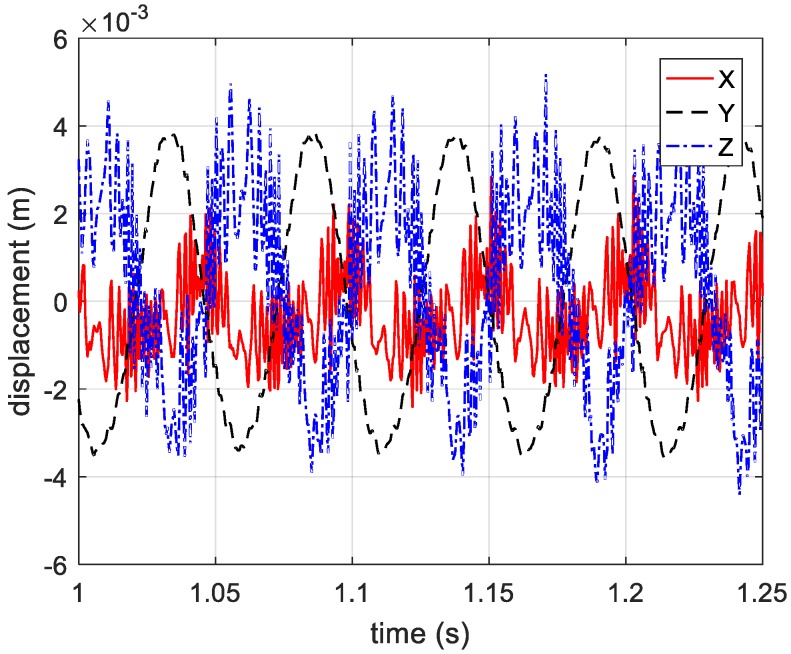
The estimated vibration components in x, y and z directions.

**Figure 21:**
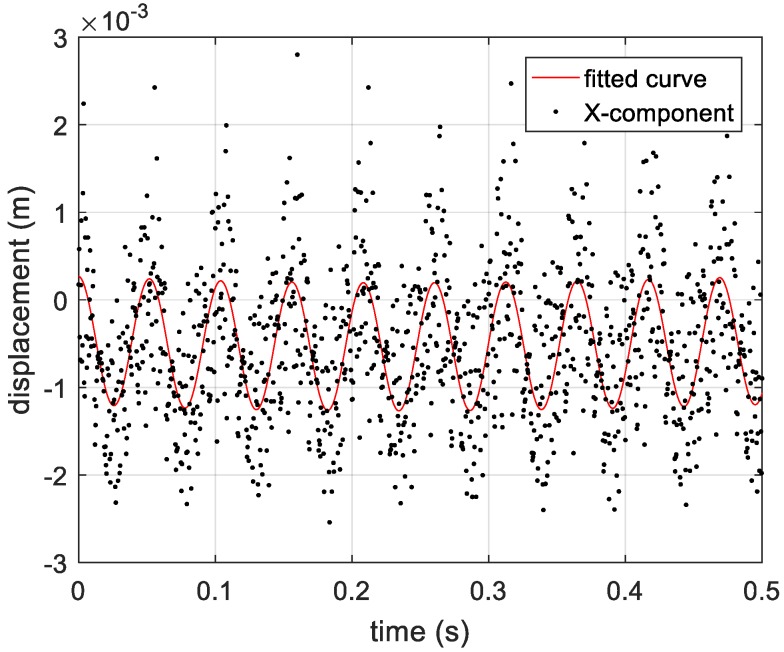
The fitted curve of vibration component in *X*-axis direction.

**Figure 22:**
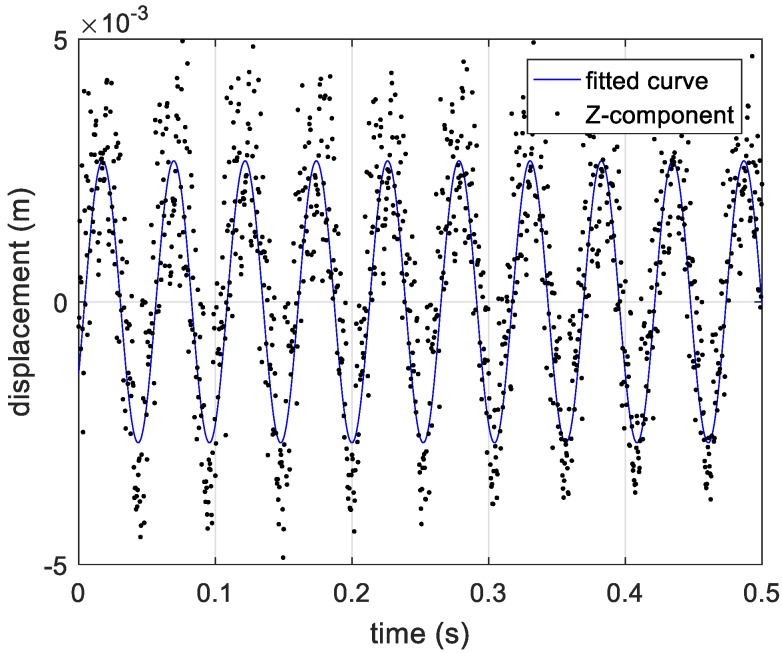
The fitted curve of vibration component in *Z*-axis direction.

**Figure 23:**
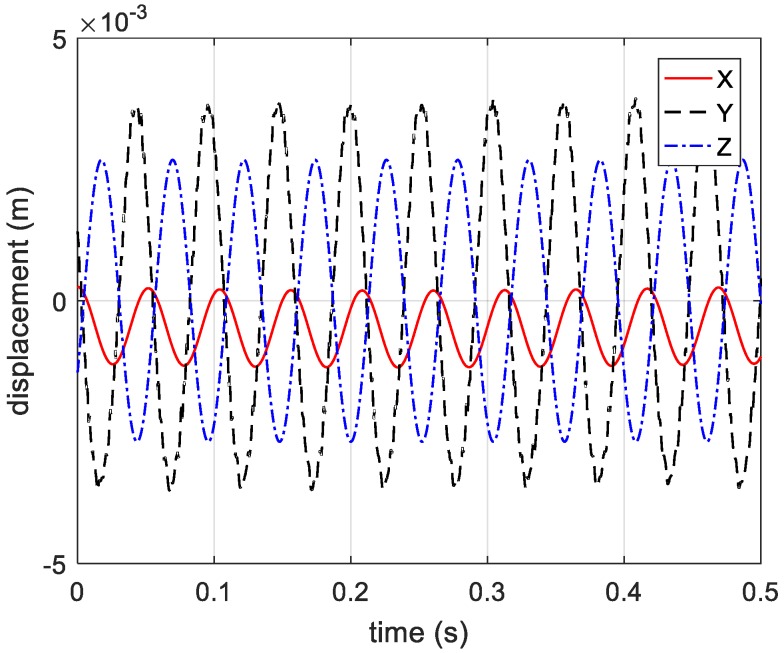
The final estimated curves of vibration components in three directions.

By compensating for the reference distance, the actual distance information between the vibrating corner reflector and three receivers is presented in Figure 19. Based on Equations (12)–(14), the estimated vibration components in axis directions are shown in Figure 20. In Figure 20, the vibration components in the *x*-axis and *z*-axis directions are not standard sine curves, but obey sinusoidal trend. Therefore, we use the sinusoidal curve fitting method to estimate the vibration components in the *x*-axis and *z*-axis directions. The root mean squared error (RMSE) values of the two fitted curves are 0.0007, and 0.0013, respectively. The estimated curves between 0.5 s to 1 s are presented in Figure 21 and Figure 22. In the end, the final estimated result of vibration components in *xyz*-axis directions are shown in Figure 23. The estimated parameters and relative errors are presented in Table 2.

### 5.2. Experimental Analysis

The estimated parameters of three-dimensional vibration in Table 2 proved the feasibility and precision of the method in this paper. The relative errors of estimated vibrating amplitude, vibrating frequency, and pitch angle are all below 5%. The relative error of the estimated azimuth angle is lower than 10%. According to the estimated parameters, we can reconstruct the trajectory of the vibrating target in a three dimensional coordinate system.

The estimated parameters are precise, except for the azimuth angle. The relative errors of the azimuth angle may be induced by the vibration testing machine. After all, the mechanical vibrating motion is inevitable to introduce errors.

Additionally, the curve of the vibration component in *x*-axis direction has the problem of phase deviation. After a series of analyses, there are two main reasons for the phase deviation. On the one hand, the error of initial distance from three receivers to the corner reflector affects the estimated coordinates. On the other, the movement of the vibration platform is not a standard harmonic motion along a straight line.

It should be noted that the coordinate system is established based on multi-channel radar, which is a relative coordinate system and is different to the absolute coordinate system. Limited by the experimental conditions, we have to adjust the angle of radar to observe the vibrating target. In this experimental scenario, it distinguishes between absolute the coordinate system and the relative coordinate system based on the radar. Thus, if we acquire the absolute coordinates, spatial coordinate transformation is necessary. On the contrary, if the multi-channel radar is placed horizontally observing vibrating target, the estimated coordinates are absolute coordinates via the method presented in this paper.

## 6. Conclusions

Three-dimensional micro-motion feature extraction and parameter estimation of the vibrating target were explored in this paper via both simulations and experiment. In this paper, we utilized the multi-channel terahertz radar system to extract three-dimensional micro-motion information. A series of simulations approved the feasibility and precision of the methods in theory. The method of phase derived range was applied to acquire the distance information from three receivers to the target. Besides, the micro-motion parameters were estimated via interference and auto-correlation procession. The performance of the method was analyzed under different SNR conditions. In addition, a 0.22 THz multi-channel radar system was adopted to verify the validity of the method. The method of curve fitting was used to estimate the vibration components in three axis directions. Meanwhile, the reasons for the errors were analyzed and explained. In the anechoic chamber using absorbing materials, the relative errors of estimated vibrating amplitude, vibrating frequency, and pitch angle were all lower than 5%. The relative error of the estimated azimuth angle was approximately 9%.

## Figures and Tables

**Table 1:** Estimated parameters of vibrating target in three dimensions.

Parameters	Vibrating Amplitude	Vibrating Frequency	Initial Phase	Azimuth Angle	Pitch Angle
Estimated values	0.0050 m	15.0037 Hz	29.9581°	29.9472°	60.1026°
Relative errors (%)	9.1615 × 10^−2^	2.4420 × 10^−2^	0.14	0.18	0.17

**Table 2:** The experimental parameters.

Parameters	Vibrating Amplitude	Vibrating Frequency	Azimuth Angle	Pitch Angle
Theoretical values	5 mm	20 Hz	10°	55°
Estimated values	4.77 mm	19.16 Hz	10.91°	55.80°
Relative errors (%)	4.60	4.20	9.10	1.45
